# Potential reduction of ventilator-associated pneumonia by a novel peristaltic feeding tube: initial evaluation of safety and efficacy in a pig model and humans

**DOI:** 10.1186/cc9620

**Published:** 2011-03-11

**Authors:** Y Avitzur, L Dayan, O Pintel, M Dayan, S McClave, P Singer

**Affiliations:** 1 Sickkids, Toronto, Canada; 2 LunGuard, Omer, Israel; 3 University of Louisville, KY, USA; 4 ICU, Rabin, Petah Tikva, Israel

## Introduction

Prevention of gastroesophageal reflux (GER) may reduce the incidence of ventilator-associated pneumonia (VAP). The aim of this study was to assess the safety and tolerance of a novel peristaltic feeding tube (PFT/LunGuard) in a pig model and healthy volunteers, and to assess its initial efficacy in preventing GER.

## Methods

The PFT is a NG feeding tube with three longitudinal balloons located at its distal end. The distal balloon is positioned 3 cm above the GE junction. The balloons are inflated/deflated sequentially in a peristaltic manner by an external monitor to prevent GER. Initially in six ventilated pigs, safety parameters including vital signs, macroscopic and microscopic inspection of the esophagus were assessed after sacrificing the animals. Prevention of GER was assessed by pH meter in one pig. In three healthy volunteers where the PFT was placed and operated for 8 hours, safety and tolerance were assessed by questionnaire given to the study subjects and by gastroscopy done pre/post PFT operation.

## Results

Each balloon was cyclically inflated for 30 seconds and then deflated. Average intermittent pressure against the esophageal wall while the balloons were inflated was approximately 30 mmHg. Visual inspection of the esophagus in both animals and humans showed no damage to the esophageal wall. Full thickness biopsies taken from esophagus under the area of the balloons as well as control biopsies taken from the proximal esophagus above showed no evidence of necrosis, ulceration, inflammation, or cell damage. Healthy volunteers reported a minimal sensation of PFT rhythmic movement at the nares and minimal discomfort in the nose and hypopharynx from the tube itself. The PFT did not interfere with normal drainage of oropharyngeal secretions. PH measurements made in the pig following injection of diluted HCl (pH = 4.0) into the distal esophagus at a maximum rate of 16 ml/second over 5 seconds showed that GER was prevented by the PFT.

## Conclusions

The PFT is safe, well tolerated and may serve to reduce risk of VAP by preventing GER in ICU patients on mechanical ventilation who are receiving enteral nutrition. Prospective clinical trials to assess PFT efficacy will be conducted.

